# The effect of combination pretreatment of donepezil and environmental enrichment on memory deficits in amyloid-beta-induced Alzheimer-like rat model

**DOI:** 10.1016/j.bbrep.2022.101392

**Published:** 2022-11-23

**Authors:** Jamileh Gholami, Sajad Sahab Negah, Arezoo Rajabian, Ehsan Saburi, Vahid Hajali

**Affiliations:** a Neuroscience Research Center, Mashhad University of Medical Sciences, Mashhad, Iran; b Department of Neuroscience, Faculty of Medicine, Mashhad University of Medical Sciences, Mashhad, Iran; c Department of Internal Medicine, Faculty of Medicine, Mashhad University of Medical Sciences, Mashhad, Iran; d Medical Genetics and Molecular Medicine Department, School of Medicine, Mashhad University of Medical Sciences, Mashhad, Iran

**Keywords:** Alzheimer's disease, Donepezil, Environmental enrichment, Combination therapy, Spatial memory, BDNF

## Abstract

Alzheimer's disease (AD) is progressive neurodegeneration known as the most common cause of dementia, and it is the sixth leading cause of death in older people. Given the promising data on the additive effect of combination therapy with donepezil (Aricept), an acetylcholinesterase inhibitor (AChEI), and regarding the similar neuronal mechanisms through which donepezil (DON) and environmental enrichment (EE) exert their enhancing effects on cognition; we asked whether simultaneous treatment with two paradigms in amyloid-beta-induced AD rats may lead to greater protection against the cognitive impairments than either treatment individually. The experimental groups consisted of Alz, sham-operated, Alz + DON, Alz + EE, and Alz + DON + EE. AD was induced by intrahippocampal injection of amyloid-beta (1–42, 6 μg), and DON was orally administrated (4 mg/kg) for 21 days. Environmental enrichment consisted of housing animals in large cages (50 × 50 × 50 cm) containing a running wheel and differently shaped objects for 21 days. Spatial learning and memory were assessed in the Morris water maze (MWM) and Real-time PCR was performed to assess the expression of brain-derived neurotrophic factor (BDNF) and M1 muscarinic acetylcholine receptor (AchM1R) within the hippocampus. Spatial memory was impaired in Alz animals, and while neither pretreatment with DON nor EE alone could significantly restore spatial memory scores in Alz rats, combination therapy was effective. BDNF expression was suppressed in Alz rats and pretreatment with DON plus EE could increase it to the saline levels. The data suggest that a cholinesterase inhibitor and cognitive stimulation can be used effectively in combination to protect cognitive loss in an AD rat model. This additive protective effect may be in part due to the augmented influence of this combination on BDNF levels and cholinergic neuronal system within the hippocampus.

## Highlights

• Spatial memory was impaired in Alzheimer's disease (AD) animals.
• Neither donepezil nor environmental enrichment (EE) alone could restore memory in AD rats.
• Combination therapy of donepezil and EE could restore the memory of AD rats to the control levels.
• BDNF expression was suppressed in AD rats and donepezil plus EE could increase it to the control levels.
• The additive effect of DON and EE may be due to the augmented influence of this combination on BDNF levels.

## Graphical abstract

**Figure:**
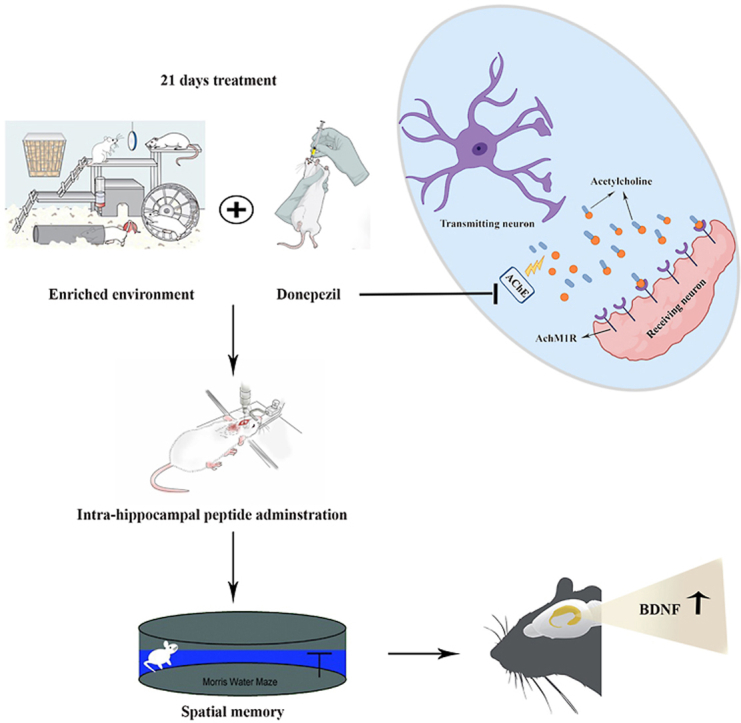

## 1 Introduction

Alzheimer's Disease (AD) is progressive neurodegeneration known as the most common cause of dementia and it is the sixth leading cause of death in older people. Current estimates suggest that 44 million people live with dementia worldwide at present and by 2030, the number of people with the disease is expected to rise to more than 70 million worldwide [1]. This profile has a lot of medical, economic, and social concerns now and in the future [2]. The accumulation of amyloid plaques and decreased levels of acetylcholine (Ach) in selectively vulnerable brain regions are the best-known pathological futures of the disease [3]. Cholinergic deficiency contributes to cognitive decline and probably behavioral symptoms of AD [4].

Donepezil (Aricept), an acetylcholinesterase inhibitor (AChEI), has for over 2 decades served as a monotherapy or in combination with NMDA-antagonist memantine for either improvement or stabilization of cognitive and functional performance of the disease [5]. It prolongs the acetylcholine's activity at the synapse by blocking its breakdown [6]. Donepezil (DON) has also been shown to decrease the amyloid plaques in the brain of humans and mice [7]. None of the AChEIs has proven more than modestly effective even at the maximum tolerated doses. There are no definitive treatments so far that completely stop the progression of AD [8]. Non-drug therapy is an alternative approach to preserve or even improve the cognitive abilities of AD [9].

Recent studies have proved the clinical and epidemiological benefits of an active lifestyle in reducing the risk of incidence or slowing the progression of cognitive disorders [10]. More advanced educational and occupational status has been consistently correlated with a lower risk of developing dementia in general and AD in particular [11,12]. Such a situation can be imitated in experimental models. Environmental enrichment (EE) is an intervention that exposes laboratory animals to new and complex stimuli due to changes in their physical environment, leading to the amplification of their sensory, cognitive, and physical stimuli. In this paradigm, the animal is placed in larger cages where there are a variety of attractive objects such as tunnels, materials used for animal nesting, toys, and running wheels [13].

The beneficial effects of EE as a potential noninvasive strategy on cognitive deficits and biochemical features of AD pathology in transgenic models have been reported [14,15]. EE increases the expression of neurotrophic factors and other signaling molecules involved in cognitive processing and exerts neuroprotective effects in AD models [[15], [16], [17]]. There are also some conflicting data implying that the effect of EE on AD pathology, neurogenesis, or cognitive performance is heterogeneous and variable [14,18]. EE could emerge as a potential non-pharmacological strategy that might affect the onset and progression of neurodegenerative diseases including AD [9,19].

The combination of different interventions for achieving superior cognitive enhancement has appeared as a promising therapeutic approach in health and disease. In the case of cognitive deficits in AD and other experimental models, the combination of DON with some treatments including piperine [20], resveratrol [21], estradiol [22], and manual acupuncture [23] has been explored. However, to date, no study has addressed the combination of DON and EE for cognitive impairments in experimentally neurodegenerative status.

Given the promising data on the additive effect of combination therapy with DON and regarding some similar neuronal mechanisms through which DON and EE exert their enhancing effects on cognition, we asked whether simultaneous treatment with two paradigms in amyloid-beta-induced Alzheimer's disease rats may lead to greater protection against the cognitive impairments than either treatment individually. Moreover, we sought to address the potential contribution of molecular mechanisms involved in learning and memory by measuring the hippocampal mRNA expression of the M1 muscarinic acetylcholine receptor (AchM1R) and brain-derived neurotrophic factor (BDNF) of the animals. Both BDNF and muscarinic cholinergic signaling have been documented to be implicated in learning and memory formation and AD pathology [[24], [25], [26], [27]].

## 2 Materials and method

### 2.1 Chemical and drugs

Amyloid-beta 1–42 was a product of Sigma Aldrich (SCP0049) and DON was purchased from Samisaz company, Mashhad, Iran. All other chemicals used in this study were of analytical grade and high purity.

### 2.2 Animals and experimental design

**Fig. 1:**
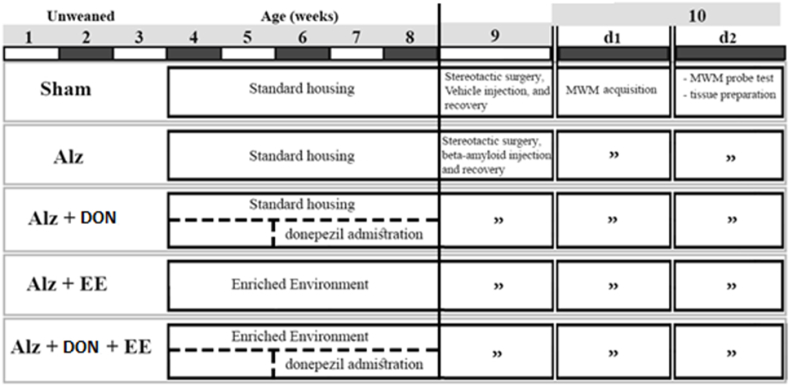
Experimental design. Animals were subjected to different treatments from week 4 to week 8 of age. Following the interventions, spatial learning and memory in Morris water maze (MWM), and hippocampal extraction were performed. Alz: Alzheimer; EE: enriched environment; DON: Donepezil. d1 and d2: day 1 and day 2. For further details see sections methods.

All experiments and animal handling were approved by the Animal Ethics Committee of Mashhad University of Medical Sciences (IR.MUMS.MEDICAL.REC.1396.683). The experimental design is depicted in Fig. 1. Male rat pups were purchased from the colony maintained by Mashhad Medical College Animal Facility.

At the age of 3 weeks, the animals were randomly assigned to one of the five experimental groups (n = 8 each) as follows:

i) Alzheimer (Alz): Rats received bilateral intrahippocampal injections of amyloid-beta (6 μg/4 μl) and were maintained in standard cages (50*30*25 cm).
ii) Sham-operated: Rats received the same volume of vehicles by the same route and were housed in standard cages.
iii) Alz + DON: Rats in standard cages treated orally with DON (4 mg/kg) for 3 weeks and then subjected to intrahippocampal injection of amyloid-beta 1–42.
iv) Alz + EE: Rats housed in larger cages (50*50*50 cm) throughout 5 weeks which were equipped with nesting materials, tunnels, ladders, shelters, houses, and toys, which were modified and rearranged weekly to increase the sense of novelty. The animals were then subjected to intrahippocampal injection of amyloid-beta 1–42.
v) Alz + DON + EE: Rats subjected to 5 weeks of the enriched environment combined simultaneously with 3 weeks of DON treatment and then subjected to intrahippocampal injection of amyloid-beta 1–42 (depicted in Fig. 1).

In all groups, behavioral tests were performed at 2 months and animals weighed 250–280 g. When behavioral tests were finished, rats were anesthetized with ketamine and euthanized by decapitation for hippocampal dissection and further analysis (Fig. 1). Animals were kept in groups of four and eight in standard and EE cages, respectively, and they had free access to food and water in all conditions and were housed in a climate-controlled room (23 °C ± 1 °C) on a 12-h light-dark cycle (lights-on 06:00–18:00 h). All cages were cleaned once a week.

### 2.3 Intrahippocampal microinjection of amyloid-beta 1-42

The animals were anesthetized with ketamine-xylazine (100-10 mg/kg, i. p.; Vibac Laboratories, Carros, France) and placed in a stereotaxic frame. Bilateral Burr holes were drilled in the skull over the CA1 region of hippocampi using the following coordinates: 3.6 mm posterior to bregma, 2.4 mm lateral to the sagittal suture, and 3.6 mm ventral to the skull surface [28]. Amyloid-beta 1–42 solution (6 μg in 4 μl PBS) or vehicle was injected bilaterally (2 μl each side) through a 27-gauge injection needle connected by a polyethylene tube to a Hamilton syringe (10 μl). The injection was delivered slowly over 10 min. The injection needle was left for an additional 60 s to maximize diffusion away from the needle tip and minimize dorsal diffusion. Before injection, the solution of Aβ was incubated for 3 days at 37 °C.

### 2.4 Spatial learning and memory in morris water maze (MWM)

The MWM was a black circular metal pool (160 cm diameter and 80 cm height) filled with water (22–24 °C)) at a depth of 50 cm. The pool was conceptually divided into four equal quadrants, and release points were labeled in each quadrant as 1, 2, 3, and 4. A hidden circular platform (10 cm diameter) was submerged 2 cm below the water surface in the center of quadrant 3. The trials were performed in a low-light room in which a variety of fixed geometric images (e.g., squares, circles, or triangles) were attached at different locations on the walls around the maze. Animal performance was recorded automatically by a video tracking system (Borj Sanat Azma), which could be traced on a computer screen. Behavioral experiments in the MWM task were accomplished on two consecutive days (Fig. 1) [29]. Each rat completed three training blocks separated by a 30-min interval during the acquisition. Each block contained a series of four successive trials of 60 s duration and three 60 s inter-trial intervals. On each trial, the animal was immersed into the water from one of the 4 quadrants of the maze and was allowed to find the hidden escape platform in 60 s (maximum time). When the animal found the platform, it was allowed to stay there for 20–30 s and returned to its home cage to wait for 20–30 s before the subsequent trial. Rats that failed to find the submerged platform within 60 s were guided to the platform. The time and distance to find the escape platform was recorded and analyzed later. A single probe trial was carried out 24 h after the last training trial to test the spatial memory in the water maze. In this trial, the animal was allowed to swim freely for 60 s without any escape platform. The percentage of the time, distance, and the number of crossing in the target quadrant (quadrant 3) was analyzed and considered the spatial memory criteria. The behavioral tasks for all groups were conducted during the same time of the lights-on phase.

### 2.5 Tissue dissection and real-time PCR

After completing behavioral experiments (Fig. 1), animals were decapitated, and both hippocampi were rapidly extracted and frozen in liquid nitrogen and then stored at −80 until further assessments. Quantitative real-time PCR (qRT-PCR) was performed to assess the hippocampal expression of AchM1R and BDNF mRNA. Total RNA was extracted by the RNeasy Mini kit (Parstous, Iran). A nanodrop spectrophotometer measured total RNA concentration at 260 nm absorbance (Thermo Fisher Scientific, Germany). According to the manufacturer's instructions, the first-strand cDNA was synthesized with 1000 ng of RNA using the cDNA Synthesis Kit (Yekta-Tajhiz; Cat: Yt4500). qRT-PCR amplification was performed with a CFX 96 Real-Time System (Roche Applied Science, USA) using Syber Green dye (Amplicon, Denmark). All qRT-PCR reactions were done in duplicate. Relative expression levels of the target genes were calculated using The 2-ΔΔCq (Livak) method by normalization to the internal control (β-actin). The sequences of the primers and the annealing temperature are shown in Table 1. *2.6. Statistical analysis*.

**Table 1:** Summary of primers used in the polymerase chain reaction.

Gene	Length (bp)	Forward: 5ʹ→3ʹ	TA (°C)
		Reverse: 3ʹ→5ʹ	
**BDNF**	122	CAGTGGCTGGCTCTCATACC	60.29
		AACAGGACGGAAACAGAACG	60.89
**Chrm1**	138	AGTCCCTCACATCCTCCGAA	60.29
		TTCTTGGTGGGCCTCTTGAC	60.89
**actinβ**	98	AAGTCCCTCACCCTCCCAAAAG	60.29
		AAGCAATGCTGTCACCTTCCC	60.89

The time and distance to find the hidden platform in the MWM training blocks in the individual groups were assessed using a repeated measures analysis. These variables between the groups were analyzed using a two-way ANOVA with repeated measures (group and block as the factors). All comparisons of data collected in the probe trials, swimming speed, and gene expression were analyzed with one-way ANOVA followed by Tukey's post hoc multiple comparison test. The values are expressed as means ± SEM, and P < 0.05 was considered statistically signiﬁcant.

## 3 Results

### 3.1 Spatial learning and memory in MWM

**Fig. 2:**
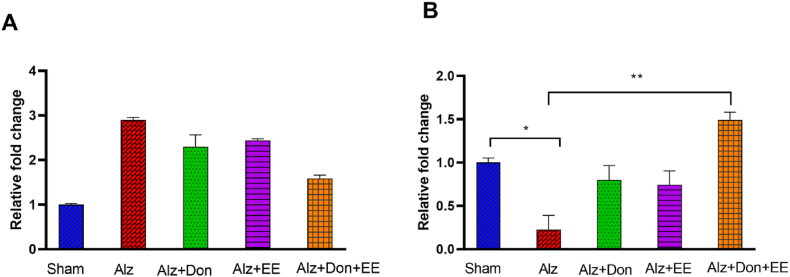
A, B, C, and D. (A and B) The spatial learning in the Morris water maze (MWM) test in sham, Alz, Alz + DON, Alz + EE, and Alz + DON + EE. Each block represents the mean latency (A) and distance traveled (B) of four consecutive trials to find the hidden platform. There were no significant differences in spatial learning ability among the groups. Data are shown as mean ± S.E.M. (two-way repeated measure ANOVA). (C) The spatial memory in MWM is shown by the percentage of the time, distance, and crossing over the target quadrant. *P < 0.05. (D) The swimming speed in MWM. Data are shown as mean ± S.E.M. (one-way ANOVA followed by Tukey test). Alz: Alzheimer; EE: enriched environment; DON: Donepezil.

In acquisition trial blocks, the repeated measures analysis revealed that animals in all individual groups successfully learned the location of the hidden platform, as revealed by the decline in escape latency and distance traveled over three subsequent blocks of training (P = 0.001). No significant difference was found among the groups in escape latency and distance traveled by analyzing two-way repeated-measures ANOVA (P = 0.912 for time and P = 0.759 for distance, Fig. 2 A and B). The spatial memory parameters in the single probe trials are shown in Fig. 2C. One-way ANOVA indicated the significant differences in the percentage of time (F (4,36) = 4.567, P = 0.004), distance (F (4,36) = 4.635, P = 0.004), and crossing (F (4,36) = 2.774, P = 0.042), in the target quadrant (Q3) among the groups. Tukey's post-hoc test revealed that Alz + DON + EE and sham groups significantly spent more time and distance in the target quadrant than the Alzheimer's animals (P < 0.05). Alz + DON + EE also crossed more times over the target quadrant than Alz animals (P < 0.05). There was no significant difference in MWM swimming speed among the five experimental groups (Fig. 2. D).

### 3.2 AchM1R and BDNF mRNA expression

**Fig. 3:**
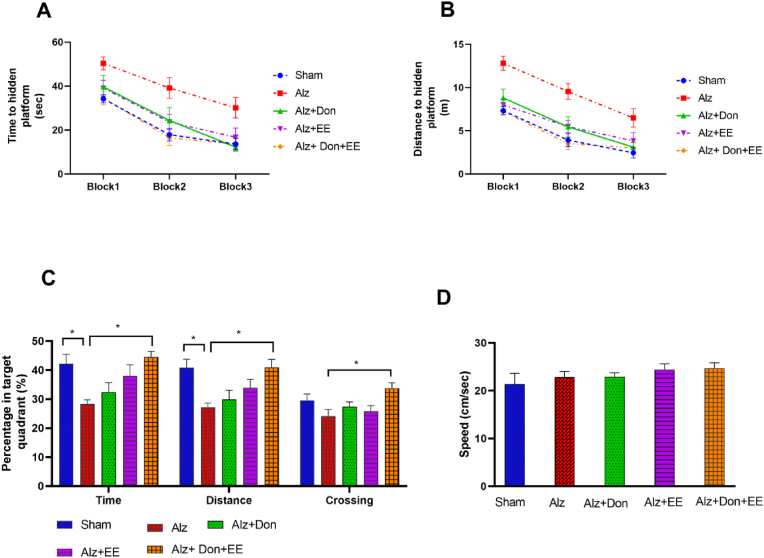
A and B. (A) The hippocampal gene expression of M1 muscarinic acetylcholine receptor (AchM1R) (A) and brain-derived neurotrophic factor (BDNF) (B) in sham, Alz, Alz + DON, Alz + EE, and Alz + DON + EE. The differences in AchM1R did not reach a significant level (P = 0.199). One-way ANOVA revealed that BDNF levels were significantly decreased in the Alz group and were returned to the normal value in the Alz + DON + EE group. *P < 0.05 and **P < 0.01. Data are shown as mean ± S.E.M. (one-way ANOVA followed by Tukey test). Alz: Alzheimer; EE: enriched environment; DON: Donepezil.

The results of AchM1R and BDNF mRNA expression in the hippocampus are depicted in Fig. 3A and B. The difference in AchM1R mRNA expression did not reach a significant level (P = 0.199, A). One-way ANOVA revealed a significant difference in hippocampal BDNF between the groups (F (4,10) = 5.838, P = 0.011, B). Tukey's post-hoc analysis showed that BDNF is decreased in Alz compared to the sham group (P < 0.05), and DON plus EE could reverse it to the vehicle value (P < 0.005).

## 4 Discussion

Based on the additive effect of the combination therapy of DON and the similar neuronal mechanisms through which DON and EE exert their enhancing effects on cognition, we hypothesized that an intervention paradigm combining DON administration as an AChEI with cognitive stimulation in Alz rats would yield greater cognitive benefits than single-domain interventions. Alz animals showed impaired function in spatial memory parameters as marked by decreased time, distance, and crossing in the target quadrant in MWM. Pretreatment with DON and EE separately could only provide slight protection from impairment in memory parameters. When animals were treated with DON and EE simultaneously, they exhibited significantly restored function as seen in saline-treated rats. Hippocampal BDNF expression was decreased in Alz animals and combination therapy of DON and EE could return it to the control levels.

Alzheimer's disease is characterized by the progressive aggregation of amyloid β-42 and hyperphosphorylated tau leading to the generation of neurofibrillary tangles, inflammation, and alterations in neurotrophic factors such as BDNF within the brain which ultimately resulted in neuronal damage, particularly in the cholinergic system [30]. Animal models with AD exhibit progressive and long-term deficits in memory function which are similar to the symptoms of sporadic AD [31,32]. Our data confirmed the previous studies showing that ICV or intrahippocampal injection of amyloid-beta causes memory impairments. However, a single injection of amyloid peptide does not express all of the pathological features of AD. There is no robust animal model that reproduces all of the characteristics of the disease, and genetic mouse models, as well as ICV or intrahippocampal injection of amyloid proteins, are the most widely applied methods for experimentally induced AD [32]. The injection of amyloid-beta into the rat brain induces pro-inflammatory reactivity, oxidative stress, and a cascade of neurotoxicity that ultimately leads to the loss of neuronal functions involved in the behavioral symptom of AD [33]. Therefore, the injection of amyloid-beta is an alternative AD animal model to transgenic animals.

Both DON and EE have been shown to improve cognitive ability scores in healthy experimental subjects as well as in experimental models with cognitive impairments [[34], [35], [36]]. However, these paradigms in the present study could not separately lead to a significant recovery of the impaired memory of AD animals. Among the several probable factors, the dose of DON used and the time course effect of EE exposure in this study may be the most important ones. By this assumption, Cavalcante et al. [37] reported that only short-term (two weeks) but not long-term (four weeks) exposure to an EE could promote the extinction of aversive memory. The cognitive benefits of DON administration in AD patients are widely suggested to be dose-response related [38,39]. In this study, we orally administrated a 4 mg/kg dose of DON once a day for 3 weeks. The administered dose of DON in animal studies varies between 0.5 and 10 mg/kg. The approved standard doses of 5 and 10 mg/day inhibit cortical AChE activity by only 20–40% but based on the dose-response relationship with DON, it was expected that increasing the dosage to 23 mg/day further increases AChE inhibition [40,41]. The dose-dependent effect of DON administration on the cholinergic system is also reported in animal studies [6,42]. Thus, the dose used in the present study may not be enough to produce separately significant improvement in the memory function of Alz animals.

The primary goal of this study was to test whether co-exposure to DON and EE is more efficient in improving cognitive deficits in AD rats than either paradigm alone. Results showed that while neither EE nor DON alone could significantly restore spatial memory scores in Alz rats, combination therapy was effective. It seems from the inspection of the data that the combination therapy was not synergistic but may be additive, with each paradigm alone producing some improvement which when combined reached significance. While, all individual groups performed the learning trials successfully; however, there was a considerable, but not significant increase in the acquisition blocks in the Alz group. Given this, and since the swimming speed was the same among the groups, it could be assumed that the observed differences in memory function may not be related to confounding factors such as specific differences in sensorimotor integration or motivation of the animals.

DON in combination with a variety of agents such as piperine [20], resveratrol [21], estradiol [22], and manual acupuncture [23] has been examined to achieve a superior benefit for neurobehavioral deficits in AD animal models. Rao et al. [21] showed that when resveratrol was combined with DON in colchicine-induced AD rats, it produced stronger stress–defense potential as proved by higher superoxide dismutase (SOD) activity. The authors conclude that resveratrol in combination with DON may have a synergic neuroprotective effect. Another study aimed to examine whether combining DON with estradiol could restore cognitive deficits in ovariectomized rats that have selective lesions in septal cholinergic neurons. It found that neither DON nor estradiol alone significantly improved the acquisition of the cognitive task in rats with cholinergic lesions. Combination therapy was efficient, however, depending on the lesion severity [22]. To date, there have been no attempts to address the potential of DON administration and EE in combination for the prevention of cognitive deficits in AD patients or experimental AD models. The partially analog studies are those that aimed to evaluate the combined benefit of cognitive rehabilitation plus DON for AD patients [43,44]. One found that the combination of cognitive rehabilitation plus a choline esterase inhibitor DON resulted in a better effect on the Mini-Mental State Examination test scores in AD patients than only drug therapy [44]. These findings suggest that the combination pattern of DON with cognitive stimulation programs is more efficacious than AChEI alone for improving cognitive and behavioral deficits in the case of CNS insults and neurodegenerative status.

Next, we sought the hippocampal expression of BDNF and AchM1R which are the key signaling molecules in memory formation and neurodegeneration [24,25]. BDNF enhances the survival, functions, and development of neuronal cells [45]. It can be found in various brain areas including the cortex, hippocampus, and basal forebrain that are essential for neurogenesis, learning, memory, and higher cognitive functions [46]. The interaction of cholinergic and BDNF signaling in memory processing has been well documented. Activation of cholinergic neurons in the medial septum increased BDNF levels [47] and depletion of cholinergic neurons in the fimbria fornix diminished BDNF mRNA in the hippocampus [48]. In healthy conditions, cholinergic transmission via muscarinic receptors seems to be involved in the regulation of BDNF synthesis to finally facilitate the consolidation of synapses in an activity-dependent manner [26]. Therefore, cholinergic neuronal activation is substantially complicated in the regulation of BDNF expression.

As amyloid-beta was injected intrahippocampal in our design, we assumed the hippocampus to be the most relevant site for assessing BDNF and AchM1R gene expression. Moreover, the hippocampus is an early brain region involved in AD pathology, revealed by amyloid-beta plaques, neurodegeneration (i.e., hippocampal atrophy), and functional impairments [49]. It is hypothesized that serum levels of BDNF are lower in AD patients than in healthy controls and subjects with mild cognitive impairment [50]. Weinstein et al. suggested that higher BDNF levels in peripheral circulation could protect older adults against AD [51]. Elevating the BDNF levels by noninvasive protocols, such as physical and cognitive stimulation, caloric-restricted diet, treatment with exogenous BDNF, and/or stimulating its receptor expression could be of potential therapeutic interest in AD [52].

It has been reported that DON increases the serum levels of BDNF in patients with AD, and BDNF upregulation is involved in the protective effect of AChEI [53]. The contribution of nerve growth factors to the enhancing effect of EE on neuronal functioning has also been well-documented [54]. A recent study demonstrated the significant role of BDNF/TrkB signaling in mediating the improving effects of EE on long-term memory and synaptic plasticity deficits induced by nerve injury in mice [55]. Therefore, we examined the potential involvement of hippocampal BDNF in memory status by measuring its mRNA expression in animals. We found that BDNF expression was suppressed in Alz rats and pretreatment with DON plus EE could increase it to the saline levels. These results are consistent with the previous findings showing a suppressed hippocampal BDNF in the amyloid-beta model of AD rats [56]. Thus, the observed additive protective effect of the DON and EE combination for memory performances of AD rats may be in part due to the augmented influence of this combination therapy on BDNF expression. A human study by Alvarez et al. [57] evaluated the effects of Cerebrolysin, DON, and combined therapy on BDNF serum levels and cognition at week 16 and week 28 after treatment of mild-to-moderate AD patients. Cerebrolysin, but not DON, increased serum BDNF at week 16, while the combination therapy enhanced it at both time points. The increasing effect of combination therapy was significantly more profound than DON and cerebrolysin groups at week 16 and week 28, respectively. These findings were associated with better cognitive improvements in AD patients, suggesting an advantage of combination therapy again for achieving the most benefits from medication in AD.

Results of AchM1R expression within the hippocampus revealed that while there was no significant difference among the groups, the Alz group showed an increase in AchM1R levels, and don, EE, and especially don + EE diminished it toward the control levels. Several neurotransmitter systems such as cholinergic, glutamatergic, adrenergic, serotonergic, and peptidergic systems are dysregulated in AD [58]. Cholinergic hypofunction in the cortex and hippocampus is one of the major hallmarks of AD pathology which is closely linked to amyloid-beta and tau pathologies [59]. Metabotropic muscarinic ACh receptors (mAChRs) as a major receptor group for ACh, have been involved in the pathophysiology of AD. The mAChR family is widely expressed in both the central nervous system (CNS) and the peripheral system. Among them, the M1 subtype makes up 50–60% of the total members of the hippocampus. The other brain areas such as the thalamus, cerebral cortex, corpus striatum, and cerebellum also express the M1 muscarinic receptor subtype [60]. AchM1R has been demonstrated to be linked to multiple functions such as cognitive functions, synaptic plasticity, neuronal excitability, and neuronal differentiation during early development [27]. Extensive evidence suggests that AchM1R signaling is implicated in AD pathology and that targeting this receptor can positively influence amyloid processing and offer disease-modifying effects in AD [25,27]. It has been reported that intracerebral injection of amyloid-beta in rats leads to the loss of cholinergic neurons and decreased Ach levels [61]. Therefore, the observed increase in AchM1R expression in the Alz group can be assumed to be related to the possible decrease in Ach concentration. However, as noted in the result section, the differences did not reach a significant level.

The studies usually address the potential benefits of DON and EE after the experimentally induced cognitive and brain function deficits (i.e., post-treatment) [20,22,62]. The pretreatment approach of DON and EE is also seen in some studies designed to apply the therapies before the experimental induction of Alzheimer-like pathology in animals [21,63]. A recent study examined the effect of DON administration at different time points (pretreatment, during ischemia, and at the onset of reperfusion) on brain pathology under conditions of cardiac ischemia/reperfusion. It found that DON treatment at all time points equally protected the brain against damages caused by cardiac ischemia/reperfusion [64]. There is also a prophylactic approach to the use of DON in human and animal studies. One reported the protective effect of pretreatment DON on cognitive deficits of patients undergoing electroconvulsive therapy [65] and another revealed that the combination of DON and procyclidine markedly protects against soman-induced seizures in rats [66]. Concerning cognitive stimulation in humans, growing evidence suggests that premorbid participation in cognitive activities reduces the risk of dementia and AD by increasing cognitive reserve [67]. In our study, we, therefore, sought to evaluate the possible preventive and prophylactic potential of combination therapy of DON and EE against cognitive impairment and molecular correlations.

## 5 Conclusion

In conclusion, these data suggest that a cholinesterase inhibitor and cognitive stimulation can be used effectively in combination to protect against cognitive loss in an AD rat model. This additive protective effect of the DON and EE combination may be in part due to the augmented influence of this combination therapy on BDNF levels and cholinergic neuronal system within the hippocampus. Given the noninvasive nature of environmental enrichment, it can be considered an efficient complementary strategy along with medication to protect against cognitive and neuronal deficits in AD patients.

## Funding

This study was supported by a grant (IR.MUMS.MEDICAL.REC.1396.683) from the research assistance of 10.13039/501100004748Mashhad University of Medical Sciences, Mashhad, Iran.

## CRediT authorship contribution statement

**Jamileh Gholami:** Data curation, Investigation, Visualization. **Sajad Sahab Negah:** Conceptualization, Writing – review & editing. **Arezoo Rajabian:** Investigation, Visualization, Software. **Vahid Hajali:** Conceptualization, Methodology, Writing – original draft, preparation, Supervision.

## Declaration of competing interest

The authors declare that they have no known competing financial interests or personal relationships that could have appeared to influence the work reported in this paper.

## Data Availability

Data will be made available on request.
